# Ezrin, more than a metastatic detERMinant?

**DOI:** 10.18632/oncotarget.27227

**Published:** 2019-11-26

**Authors:** Victoria Hoskin, Abdi Ghaffari, Bruce E. Elliott

**Affiliations:** Abdi Ghaffari, Victoria Hoskin and Bruce Elliott: Division of Cancer Biology and Genetics, Queen’s Cancer Research Institute, Ontario K7L 3N6, Canada; Department of Pathology and Molecular Medicine, Queen’s University, Kingston, Ontario K7L 3N6, Canada

**Keywords:** ezrin, metastasis, breast cancer, therapeutic target

**Figure 1:**
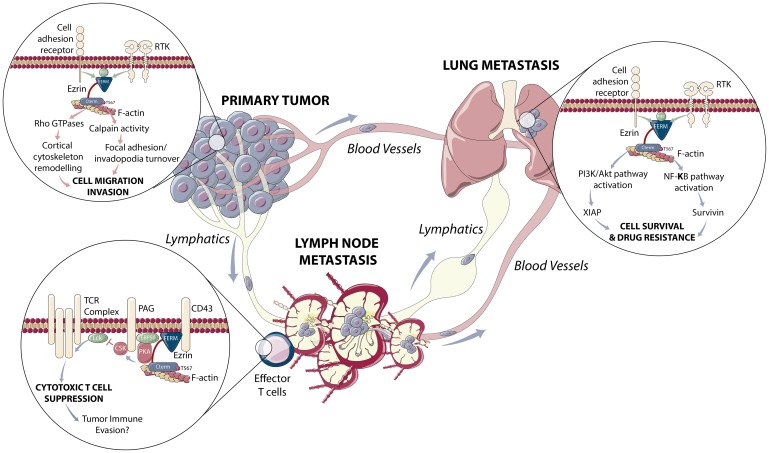
Role of ezrin in cancer metastasis and drug resistance. Ezrin is known to promote cancer progression through various molecular mechanisms, as highlighted in the signaling insets. Ezrin facilitates cell migration and invasion through its roles in focal adhesion and invadopodia turnover and cytoskeletal remodeling at the primary tumor site to promote metastasis to lymph nodes; where it contributes to cytotoxic T cell suppression and immune evasion through a PKA-CSK-LCK signaling pathway associated with the T cell receptor; and to more distant organ sites such as the lung were it contributes to cell survival and drug resistance through effects on NF-κB and PI3K/AKT signaling pathways. These multifaceted roles for ezrin in cancer makes it an attractive therapeutic target, where its inhibition may suppress metastasis, promote anti-tumor immunity and enhance cytotoxic drug sensitivity. Illustration was created by Designs That Cell.

Metastasis is responsible for the vast majority of all cancer-related deaths [1]. While many modern systemic therapies are unsuccessful at treating metastatic disease [1], recent advances in our understanding of the metastatic process and its regulators are revealing potential novel therapeutic targets. Ezrin, a member of the ERM (Ezrin-Radixin-Moesin) family of cytoskeleton adaptor proteins, is frequently over-expressed in invasive cancers and is associated with poor overall survival [2]. Ezrin controls pro-metastatic phenotypes including cell migration and invasion [3, 4], and is known to regulate multiple aspects of the metastatic process, such as angio- and lymphangiogenesis [5] and distant organ seeding [4] (Figure 1) - all of which make this molecule an intriguing anti-metastatic target and prognostic biomarker.

Our group has recently demonstrated the efficacy of a small molecule inhibitor of ezrin in blocking metastatic progression, using a novel intravital imaging model of lymph node (LN) metastasis [3]. LNs are a common site of metastasis for many invasive epithelial cancers, including breast and prostate, both of which show a preference for lymphatic dissemination [6]. Clinically, LN metastasis is one of the strongest indicators of disease recurrence in breast cancer patients [6]. However, the uncertainty in reliably predicting which LN positive patients will benefit from more aggressive systemic therapies versus those who will remain distant metastasis-free represents a major clinical challenge. Thus, the need for more robust predictive markers of disease relapse, coupled with the need for better preclinical model systems to advance our knowledge of LN metastasis, is critical. With our intravital model, we show that systemic treatment with the small molecule ezrin inhibitor, NSC668394, is capable of reducing cancer cell migration, one of the hallmarks of metastasis, within tumor-draining LNs (TDLNs) [3]. Furthermore, we demonstrate that reducing ezrin-mediated cell motility within TDLNs also impedes cancer cells from spreading to more distal axillary LNs and to the lungs [3]. This model therefore reveals that targeting ezrin can inhibit cancer cell dissemination through the lymphatic system and provides evidence that cancer cells within LNs can serve as reservoirs for subsequent spread to distant organ sites. Given the importance of a patient’s LN status in predicting risk of relapse, there is considerable value in utilizing preclinical models of LN metastasis to test the efficacy of anti-metastatic agents in reducing node-positive disease and preventing further dissemination.

LN status remains a gold standard for assessing relapse risk, however not all node-positive breast cancer patients will relapse and greater than 25% of apparent LN-negative patients eventually develop recurrence [7]. This statistic highlights the limitations of LN status and the need for markers that will better stratify patients who are at risk of relapse. In a locally accrued cohort of breast cancer patients, we recently show that ezrin is an independent marker of recurrence in both LN positive and high risk LN-negative breast cancer patients [3], suggesting that the prognostic power of ezrin is not restricted to the LN positive patient population and may potentially be a more robust marker for disease recurrence. Ezrin expression is also shown to increase from primary tumors to LN metastasis in matched tissues from the same patient [3]; this finding in combination with results from our intravital model demonstrates that ezrin activity confers a metastatic advantage for tumor cells and remains biologically active at metastatic sites.

Interestingly, targeting ezrin in our LN metastasis model principally affected metastatic dissemination with minimal changes to primary tumor growth [3], consistent with previous knockdown studies on ezrin [4]. This has important implications relating to the development of novel therapies to target microscopic disease as well as for new combination treatments. For instance, many first-line systemic therapies are aimed at reducing primary tumor burden with the goal of preventing and/or reducing cancer cell spread. However, few of these treatments are effective at eliminating occult or microscopic metastases, i.e. clinically undetected, slow growing/quiescent cancer cells which have already metastasized by the time of initial treatment [1]. Indeed, in a neoadjuvant preclinical model, some cytotoxic treatments have been shown to actually promote metastatic dissemination [8]. It is therefore important to consider combination strategies that effectively target both the primary tumor and micro-metastatic cell populations. Our preclinical data suggest that ezrin-targeted therapy is effective at reducing the burden of metastasis in the LN and lungs [3]. Furthermore, given ezrin’s role in promoting survival of metastatic cells [4], targeting ezrin, or similarly acting molecules, may also provide additional anti-metastatic benefit in combination with anti-tumor/cytotoxic therapies [9].

Cancer cells within LNs are also capable of manipulating host immune cell activity to create a microenvironment conducive to tumor outgrowth. In addition to tumor-cell specific expression, ezrin is also expressed by cytotoxic T-lymphocytes (CTLs) and is implicated in regulating T-cell activity (Figure 1). In this context, ezrin functions as an A-kinase Anchoring Protein (AKAP) to promote PKA-mediated suppression of CTL activation [10]. Studies have shown that disrupting ezrin localization to the T cell receptor complex prevents cAMP/PKA-mediated inhibition of CTL activation [10]. As breast cancer is considered to be less immunogenic than melanoma and non-small cell lung carcinomas, new strategies to enhance immune cell activity are needed to improve patient response to immunotherapy. Preliminary evidence from our single-cell resolution intravital model shows increased tumor cell/T-cell engagement and number of active T cells (CD69^pos^/CD62L^neg^) in TDLN of mice treated with the ezrin inhibitor, suggesting that ezrin-targeted therapy, in addition to inhibiting metastasis, may also enhance host T cell anti-tumour activity (unpublished results).

In summary, ezrin is involved in multiple aspects of metastasis [4, 5]. Our recent clinical studies provide valuable information regarding the prognostic potential of ezrin in high risk LN-negative breast cancer patients [3] and possibly in other cancer subtypes. Furthermore, there is strong preclinical evidence demonstrating ezrin as a targetable biomarker of metastasis and convincing rationale for investigating the effect of combining ezrin-targeted therapy with chemotherapeutic or immunotherapeutic agents.
